# Supplementary material for: Biomarker Testing in Older Patients Treated for an Advanced or Metastatic Non-Squamous Non-Small-Cell Lung Cancer: The French ESME Real-Life Multicenter Cohort Experience
Source: Cancers (Basel). 2021 Dec 24;14(1):92. doi: 10.3390/cancers14010092 (PMC8750267; doi:10.3390/cancers14010092)
Supplement: Supplementary file 1 [file cancers-14-00092-s001.zip › cancers-1461935-supplementary.pdf]

Supplementary data

Biomarker testing at aNSCLC diagnosis according to age (≥ 75 years old , and ≥ 80 year old respectively compare to < 70 years old)

Biomarker testing was defined as, at least one molecular alteration and/or PD-L1 testing performed within 1 month before or 3 months after the aNSCLC diagnosis. « Molecular testing only » meant testing for at least one molecular alteration excluding the search for PD-L1 status.

Supplementary analysis with increased age cutoff for old patients (≥ 75 years old and ≥ 80 year old respectively), showed no significant difference with younger patient in biomarker testing at aNSCLC diagnosis, neither between patients aged ≥ 75 years and their younger counterparts (adjusted odd ratio 1.03, 95%CI 0.89-1.19, p=0.6909), nor between patients aged ≥ 80 years and younger (adjusted odd ratio 0.97, 95%CI 0.80-1.18, p=0.7486).

Biomarker testing at aNSCLC diagnosis N = 9748	< 75 N = 8107	≥ 75 N = 1641	p
Yes	5234 (65%)	1051 (64%)	0.6909
Molecular testing only	3823 (47%)	797 (49%)	
PDL1 testing only	177 (2%)	29 (2%)	
Molecular & PDL1 testing	1233 (15%)	225 (14%)	
Missing	1	0	

Biomarker testing at aNSCLC diagnosis N = 9748	< 80 N = 8949	≥ 80 N = 799	p
Yes	5774 (65%)	511 (64%)	0.7486
Molecular testing only	4228 (47%)	392 (49%)	
PDL1 testing only	193 (2%)	13 (2%)	
Molecular & PDL1 testing	1532 (15%)	106 (13%)	
Missing	1	0	
